# Distribution of sialic acid receptors and influenza A virus of avian and swine origin in experimentally infected pigs

**DOI:** 10.1186/1743-422X-8-434

**Published:** 2011-09-08

**Authors:** Ramona Trebbien, Lars E Larsen, Birgitte M Viuff

**Affiliations:** 1 Division of Veterinary Diagnostics and Research, National Veterinary Institute, Technical University of Denmark, Bülowsvej 27, 1790 Copenhagen V, Denmark; 2 Department of Veterinary Disease Biology, Faculty of Life Sciences, University of Copenhagen, Dyrlægevej 88, 1870 Frederiksberg C, Denmark

## Abstract

**Background:**

Pigs are considered susceptible to influenza A virus infections from different host origins because earlier studies have shown that they have receptors for both avian (sialic acid-alpha-2,3-terminal saccharides (SA-alpha-2,3)) and swine/human (SA-alpha-2,6) influenza viruses in the upper respiratory tract. Furthermore, experimental and natural infections in pigs have been reported with influenza A virus from avian and human sources.

**Methods:**

This study investigated the receptor distribution in the entire respiratory tract of pigs using specific lectins *Maackia Amurensis *(MAA) I, and II, and *Sambucus Nigra *(SNA). Furthermore, the predilection sites of swine influenza virus (SIV) subtypes H1N1 and H1N2 as well as avian influenza virus (AIV) subtype H4N6 were investigated in the respiratory tract of experimentally infected pigs using immunohistochemical methods.

**Results:**

SIV antigen was widely distributed in bronchi, but was also present in epithelial cells of the nose, trachea, bronchioles, and alveolar type I and II epithelial cells in severely affected animals. AIV was found in the lower respiratory tract, especially in alveolar type II epithelial cells and occasionally in bronchiolar epithelial cells. SA-alpha-2,6 was the predominant receptor in all areas of the respiratory tract with an average of 80-100% lining at the epithelial cells. On the contrary, the SA-alpha-2,3 was not present (0%) at epithelial cells of nose, trachea, and most bronchi, but was found in small amounts in bronchioles, and in alveoli reaching an average of 20-40% at the epithelial cells. Interestingly, the receptor expression of both SA-alpha-2,3 and 2,6 was markedly diminished in influenza infected areas compared to non-infected areas.

**Conclusions:**

A difference in predilection sites between SIV and AIV virus was found, and this difference was in accordance with the distribution of the SA-alpha-2,6 and SA-alpha-2,3 receptor, respectively. The results indicated that the distribution of influenza A virus receptors in pigs are similar to that of humans and therefore challenge the theory that the pig acts as a mixing vessel between human and avian influenza viruses. Furthermore, it was shown that AIV prefers to infect alveolar type II epithelial cells in pigs. This corresponds with findings in humans emphasising the resemblance between the two species.

## Background

The natural reservoir of influenza A virus (infAv) is considered to be aquatic birds because all known subtypes (H1-H16 and N1-N9) of infAv have been isolated from waterfowl [1]. InfAv can, however, infect many other mammalian species, including humans, swine, horses, ferrets, and sea mammals [1-3]. There are several specific swine influenza A virus (SIV) subtypes (H1N1, H1N2 and H3N2) circulating in the pig populations in Europe, including Denmark [4-6]. The Danish SIV subtype H1N2 differs from the European SIV H1N2 subtypes, in that it is a re-assortment between two circulating Danish SIV strains of the subtypes H1N1 and H3N2. The first known Danish H1N2 isolate occurred in 2003 and is therefore a relatively new strain in Denmark [7].

It has been described that pigs have receptors for both human and avian strains of influenza A viruses in the upper respiratory tract and therefore are susceptible to infections by both [8,9]. Based on this finding, it has been proposed that pigs can act as a mixing vessel when infected by both a human and avian influenza A virus (AIV) strain to make a new reassorted virus with zoonotic and even pandemic potential. In recent years, however, there have been examples of infAv crossing the species barrier without the involvement of pigs [10-12]. Infections with infAv are initiated by interactions between virus haemagglutinin and sialic acid (SA) molecules on target cells. AIV strains prefer SA-α-2,3-terminal saccharides whereas human and swine influenza virus strains prefer SA-α-2,6-terminal saccharides as receptors [9,13-16]. A few studies have shown that the epithelial cells of the upper respiratory tract of pigs express both receptors [8,9]. However, recent studies have shown a more variable distribution of the specific receptors in the deeper lung areas whereas in the trachea the SA-α-2,6-terminal saccharides are abundant [17,18]. It has been described that after infections with AIV in pigs and humans the virus has shifted receptor specificity from SA-α-2,3 to SA-α-2,6 as a part of the adaptation to the new host by the virus. This shift in receptor specificity has been linked to specific amino acid substitutions in the HA molecule [19-21], but the exact determinants of the host specificity of infAv have not been fully elucidated.

Specific lectins have been the chosen method for detecting SA receptors. The *Sambucus Nigra *(SNA) lectin is specific for SA-α-2,6 bindings and the *Maackia Amurensis *(MAA) lectin is specific for SA-α-2,3 bindings of the SA molecules. In order to detect SA-α-2,3-terminal saccharides it is necessary to use two isoforms of MAA lectin: MAAI and II because the two isoforms are different in the way they recognise the inner sugar structures of SA-α-2,3 [22-24]. A more thorough investigation of the receptor distribution in the respiratory tract of pigs would give a more nuanced picture of the infection dynamic of different infAv in pigs. This, together with investigation of the predilection site of different infAv in the respiratory tract tissue, would enable us to improve our understanding of the mechanisms of infection regarding pathogenesis and host range determination.

The aim of the study was to investigate the tissue and cell predilection sites of avian and swine influenza A viruses, respectively, and SA-α-2,3/2,6-terminal saccharide receptor distribution in the respiratory tract of pigs by the use of immunohistochemical methods and lectin staining.

## Methods

### Animals

Landrace/Yorkshire (LY) pigs two months of age were used for the experiments. The pigs were bred for experimental use, and were free from the important pathogens: porcine circovirus 1, porcine circovirus 2, porcine reproductive and respiratory syndrome virus, swine influenza A virus, porcine respiratory coronavirus, and Mycoplasma [25]. All pigs were tested negative for SIV and AIV before the experiments and were examined and shown negative for antibodies against SIV and AIV. The different groups were housed in separated isolation units. A total of 21 pigs were included in this study of which 8 pigs were inoculated with influenza virus (4 with SIV and 4 with AIV) (in total 8 influenza virus positive pigs). Furthermore, 4 pigs were mock inoculated and one pig was not inoculated (see below). Additionally, tissue samples were included for the lectin staining from 8 pigs which had recovered from SIV (H1N1 and H1N2) infections 8 weeks earlier. The infection and cease of infection was diagnosed by both virus and antibody detection (in total 13 influenza negative pigs).

Tissues from 8 chickens were included as positive control tissues for the lectin stainings [26].

The study was carried out in strict accordance with Danish legislation on animal experiments (LBK nr 1306 - 23/11/2007) and EU regulations on the use of laboratory animals for research.

### Experimental design

#### SIV infected pigs

Two pigs were inoculated intranasally at post inoculation day (PID) 0 with 4 ml of A/swine/Denmark/19126/1993 (H1N1) 3^rd ^passage grown in primary swine kidney cells containing a titer of 10^5.7 ^tissue culture infectious dose 50% (TCID50) per ml and euthanized PID 4.

Two pigs were inoculated intranasally at PID 0 with 4 ml (10^5.9 ^TCID50 per ml) of A/swine/Denmark/10074/2004 (H1N2) 3^rd ^passage and euthanized PID 4.

#### AIV infected pigs

At PID 0, four pigs were inoculated with 4 ml intranasally and 4 ml intratracheally (titer: 10^8 ^egg infectious dose 50% (EID50) per ml) of A/duck/Denmark/65472-26/2003 (H4N6) 3^rd ^passage propagated and harvested from allantoic fluid of embryonated chicken eggs. The pigs were inoculated both intranassally and intratracheally to increase the chance of successful infection with AIV. Two pigs were euthanized on PID 4 and 8, respectively.

#### Negative controls

One pig was not inoculated and was housed together with the AIV infected pigs (sentinel). The pig was euthanized PID 8. Four pigs were included as uninfected (infAv free) controls. The pigs were inoculated PID 0 with mock consisting of pure allantoic fluid from embryonated chicken eggs, and received 4 ml intranasally and 4 ml intratracheally. Two of the pigs were euthanized PID 4 and two pigs were euthanized PID 8.

### Necropsy

Euthanization of the pigs was performed using pentobarbiturat (50 mg/kg) intraveneous following exsanguination by cutting arteria axillaris. Immediately after killing, tissue samples from the following organs were collected: ciliated nose epithelium, palatine tonsil, trachea (section from the middle part), *lnn. tracheobronchialis*, and nine lung tissue samples from the following areas: lu1: cranioventral part of the left cranial lobe; lu2: caudoventral part of the left cranial lobe; lu3: dorsal part of the left cranial lobe; lu4: ventral part of the right cranial lobe; lu5: middle part of the right cranial lobe; lu6: dorsal part of the right cranial lobe; lu7: middle part of the right middle lobe; lu8: middle part of the accessory lobe; and lu9: middle part of the right caudal lobe.

The tissue from the 8 chickens included trachea, lung and intestines and were treated as described for the pig tissues.

The tissues were fixed in 10% PBS-buffered formalin for 24-30 hours, washed with demineralized water (dH_2_O), and transferred to 70% ethanol where the tissues were stored for a maximum of 14 days. The tissues were paraffin embedded, cut into 3 μm sections and mounted on superfrost + glass slides (Menzel-Gläser). Prior to immunohistochemistry (IHC) and lectin staining, the tissue sections were deparaffinised in xylene, rehydrated through graded alcohols and washed three times in Tris buffered saline (TBS), pH 7,6 (0,05 M Tris-HCl, 0,15 M NaCl).

All tissue sections were evaluated in double for IHC with infAv antibody. Tissues from SIV infected pigs were evaluated with a polyclonal antibody against SIV and a polyclonal antibody against infAv (see below). Tissues from AIV, mock and non-inoculated animals were evaluated twice with a polyclonal antibody against infAv (see below). For the first evaluation of the lectin staining, all lung sections and sections of nose and trachea were evaluated. For the second evaluation of lectin staining one representative section from cranial (lu2) and caudal (lu9) lung lobes, respectively, were picked for final assessments together with sections from nose and trachea.

All microscopic evaluations of IHC and receptor staining were performed with an Olympus BX50 microscope and photographs were taken with an Olympus DP70 camera using the photographic software Olympus DP-soft.

### Receptor staining

DIG glycan differentiation kit from Roche (cat. no. 11 210 238 001) was used for detection of the SA-α-2,6-terminal saccharide receptor. In brief, blocking was performed with the blocking reagent for 30 minutes at room temperature, followed by three washes in TBS. For the detection of SA-α-2,6-terminal saccharides, digoxigenin (DIG) labelled *Sambucus nigra *(SNA) lectin, were used. The SNA lectin was diluted 1:1000 in Buffer 1 and incubated overnight at 4°C. This was followed by three washes in TBS before incubation for one hour at room temperature (RT) with anti-digoxigenin alkaline phosphatase diluted 1:1000 in TBS. The sections were washed three times in TBS and developed for 5 minutes at RT with Nitroblue tetrazolium/5-bromo-4-chloro-3-indolyl-phosphate (NBT/BCIP) diluted 1:50 in Buffer 2 revealing a dark blue staining. A final wash was performed three times with TBS before the sections were mounted with glycergel and evaluated in microscope.

The same procedure was used for staining of the SA-α-2,3-terminal saccharide receptors except for incubation with biotinylated *Maackia amurensis *(MAA) I lectin (cat. no. B-1315 Vector laboratories) diluted 1:4000 in Buffer 1 or biotinylated MAA II lectin (cat. no. B-1265 Vector laboratories) diluted 1:2000 in Buffer 1 overnight at 4°C. Afterwards the sections were washed three times with TBS and incubated for one hour at RT with streptavidine alkaline phosphatase (cat. no. SA-5100 Vector Laboratories) diluted 1:200 in TBS.

### Receptor scoring

To evaluate the receptor distribution, a scoring system was developed. The lectin staining was evaluated in categories of percent of the lectin positive distribution on the epithelial cells in nose, trachea, bronchi, bronchioles and alveoli. The categories were; 1: 0-20%, 2: 20-40%, 3: 40-60%, 4: 60-80% and 5: 80-100%. Double blinded evaluation of the lectin staining was performed. A weighted kappa test was calculated after evaluation [27].

To generate the score for the lectin staining the whole tissue section (approx. 1 by 2 cm) was evaluated. Tissue sections from infAv infected animals containing both consolidated and unconsolidated areas were evaluated separately in the two parts whereby the same section could obtain two different scores.

### Immunohistochemical staining of SIV antigen

The tissue was treated for ten minutes with 0.018% protease in TBS (Protease, Sigma cat. no. P-8038) at RT for antigen retrieval. The sections were then washed for 5 minutes with 4°C cold TBS followed by washing in TBS at RT for 5 minutes. Blocking was performed against endogenous peroxidase activity in 3% H_2_O_2 _for 5 minutes, followed by a wash for 5 minutes in dH_2_O and three washes in TBS. Blocking against nonspecific protein binding was performed using 5% normal pig serum in TBS for at least 10 minutes at RT. The primary antibody, polyclonal rabbit anti-SIV, was made by immunization of rabbits with the isolate: A/swine/Denmark/4744/1981 (H1N1). The antibody was diluted 1:1000 in 5% normal pig serum in TBS and applied to the sections which were incubated overnight at 4°C. The sections were washed three times in TBS before incubation for 30 minutes at RT with swine anti-rabbit antibody (DAKO cat. no. Z196) diluted 1:100 in 5% normal pig serum in TBS. After wash in TBS, the sections were incubated with peroxidase antiperoxidase (PAP), rabbit (DAKO cat. no. Z113) diluted 1:100 in 5% normal pig serum in TBS for 30 minutes at RT. The sections were washed in TBS and then developed using DAB+ (DAKO cat. no. K3468) for 40 minutes at RT revealing a brown staining. Finally, the sections were washed in TBS and counterstained with Mayers hematoxylin for 10 seconds before mounting with glycergel.

### Staining of SIV and AIV antigens

Antigen retrieval was performed by microwave boiling of the slides in Tris/EDTA buffer (10 mM Tris, 1 mM EDTA, pH 9). The slides were left in the warm buffer for 15 minutes before wash in TBS. Blocking was performed for 10 minutes in 5% normal pig serum in TBS followed by incubation overnight at 4°C with goat anti infAv antibody (cat. no. OBT1551 Serotec, immunogen: InfAv, strain USSR (H1N1)) diluted 1:5000 in 5% normal pig serum in TBS. After wash in TBS the sections were incubated with biotinylated rabbit anti-goat antibody (cat. no. E0466 DAKO) diluted 1:200 in 5% normal pig serum in TBS for 30 minutes at RT. The sections were then washed in TBS and incubated with streptavidin - alkalic phosphatase conjugate (cat. no. SA-5100 Vector Laboratories) diluted 1:400 in 5% normal pig serum in TBS for 30 minutes at RT. Development was performed in Fast Red substrate (KemEnTec diagnostics cat. no. 4210) for 20 minutes at RT revealing a red staining, followed by three washes in TBS before counterstaining in Mayers hematoxylin and mounting with glycergel.

### Double staining of SIV antigen and cytokeratin

The double immunohistochemistry clarifies if the infAv antigen positive cells protruding from the alveolar wall or situated in the alveolar lumen were alveolar type II epithelial cells or alveolar macrophages. Double stainings were performed as described before by Viuff *et al. *[28] except the use of other antibodies. Breifly, antigen retrieval was performed with 0.018% protease for 30 minutes at RT followed by blocking with 5% normal pig serum in TBS. The sections were then incubated with polyclonal rabbit anti-SIV diluted 1:1000 in 5% normal pig serum in TBS for one hour at 37°C, followed by incubation with biotinylated swine anti-rabbit (DAKO cat. no. E0431) diluted 1:300 in 5% normal pig serum in TBS for 30 minutes at RT. The sections were then incubated with streptavidin-β-galactosidase (Roche cat. no. 11112481001) diluted 1:1000 in 5% normal pig serum in TBS for 30 minutes at 37°C. The staining was performed using X-Gal substrate (Histomark, Kirkegaard & Perry Laboratoriums cat. no. 54-13-00) for 30 minutes at RT. Positive stainings were blue. The sections were left in TBS overnight and then incubated for 1 hour at 37°C with monoclonal mouse anti-human cytokeratin antibody (DAKO cat. no. M0821) diluted 1:50 in 5% normal pig serum in TBS. This was followed by incubation for 30 minutes at RT with rabbit anti-mouse (DAKO cat. no. Z259) diluted 1:25 in 5% normal pig serum in TBS. The sections were then incubated at RT for 30 minutes with alkaline phosphatase-anti-alkaline phosphatase (APAAP), mouse, complex (DAKO cat. no. D651) diluted 1:50 in 5% normal pig serum in TBS and afterwards developed with Fast Red (KemEnTec diagnostics cat. no. 4210) for 60 minutes at RT revealing a red staining. The sections were finally counterstained with Mayers hematoxylin and then mounted with glycergel. Double stained cells appeared purple.

### Double staining of AIV antigen and cytokeratin

The procedure was as described above except the use of goat anti-infAv antibody (cat. no. OBT1551 Serotec, infAv, strain USSR (H1N1)) diluted 1:1000 in 5% normal pig serum in TBS followed by incubation for 30 minutes with biotinylated rabbit anti-goat antibody (cat. no. E0466 DAKO) diluted 1:200 in 5% normal pig serum in TBS. The sections were then incubated with streptavidin-ß-galactosidase (Roche cat. no. 11112481001) diluted 1:1000 in 5% normal pig serum in TBS for 30 minutes at 37°C and developed for 60 minutes with X-Gal substrate (Histomark, Kirkegaard & Perry lab cat. no. 54-13-00).

In the double staining protocols, control sections were included in the form of lung tissue sections from mock-inoculated control pigs and only cytokeratin was demonstrated in these sections. Likewise, only single staining was obtained when either of the primary antibodies was omitted when staining lung sections from infAv infected pigs.

## Results

### Clinical signs

There were no visible clinical signs during the experiment in the AIV and SIV inoculated pigs. In the SIV inoculated pigs mildly increased body temperature (between 0.1 and 1°C) was recorded on day 1 only.

### Macroscopic changes

Pigs inoculated with SIV H1N1 and euthanized PID4 had few lobular consolidated areas in the cranial lung lobes. In the left cranial lobe and the middle lobe the consolidated areas constituted < 10% of the lobes. The SIV H1N2 infected pigs, likewise euthanized PID4, had more widespread consolidations in the lung lobes compared to the H1N1 infected pigs. In the left cranial lobes an average area of approximately 80% was consolidated and approximately 40% of the middle lobe was consolidated. The accessory lobe had an average of 20% affected areas. The right and left caudal lobes had few lobular consolidations in the cranial parts. The right cranial lobes were only sparsely affected.

In general pigs inoculated with AIV H4N6 had no gross pathological changes except for one pig euthanized PID 4 where minor lobular consolidations were present in the right cranial lobe, right middle lobe, and left cranial lobe.

Mock and non-inoculated pigs and pigs which had recovered from SIV infections 8 weeks before had no gross pathological changes.

### Microscopic findings

Pigs inoculated with SIV H1N2 showed more affected areas compared to SIV H1N1 inoculated pigs. The consolidated areas had a lobular distribution with bronchitis and bronchiolitis and to a lesser extent alveolitis in the most severely affected areas. Hyperplasia of the epithelial cells was seen in the affected bronchi and bronchioles and many of the bronchial epithelial cells had lost their cilia. Cellular exudate consisting of neutrophil granulocytes and few mononuclear cells was seen in the lumen of bronchi and bronchioles and sometimes in alveoli. In some animals there was peribronchial and peribronchiolar infiltration with mononuclear cells and in the most severely affected areas interstitial oedema was observed. Pigs inoculated with AIV H4N6 had only few affected areas, especially in comparison to pigs inoculated with the SIV subtypes H1N1 and H1N2. Consolidations were seen in a lobular distribution in affected areas. A sparse cellular exudate was occasionally present in the lumen of alveoli and bronchioles in affected areas. Mock and non-inoculated pigs as well as pigs which had recovered from SIV infections 8 weeks before had no histopathological changes.

### Receptor staining

#### SA-α-2,6-terminal saccharides (SNA lectin)

**Figure 1:**
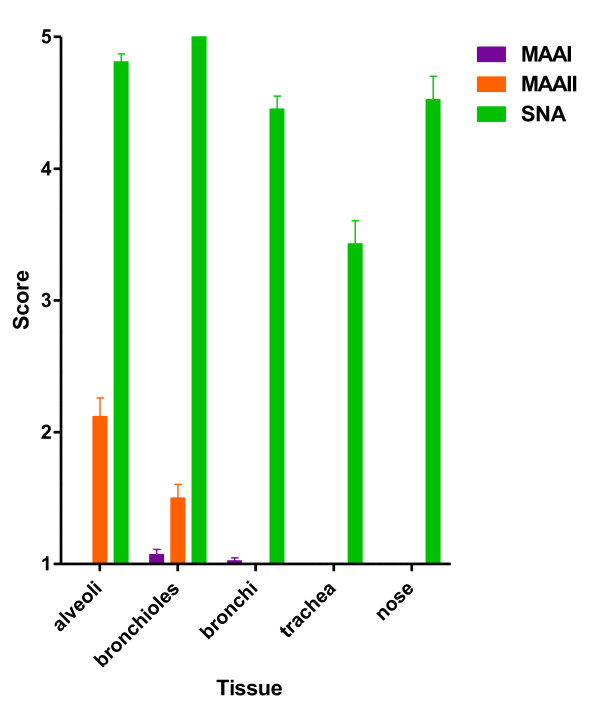
**The distribution of lectin staining in the respiratory tract of pigs**. Diagram showing the score distribution of the three lectins MAAI (SA-α-2,3-terminal saccharides), MAAII (SA-α-2,3) and SNA (SA-α-2,6) at the epithelial cells of alveoli, bronchioles, bronchi, trachea, and nose. (Score 1: 0-20%, 2: 20-40%, 3: 40-60%, 4: 60-80% and 5: 80-100%). The diagram is based on an average of both infAv positive (n = 8) and infAv negative (n = 13) pigs. Only scoring data from unconsolidated areas of the tissue sections is included in the figure.

**Figure 2:**
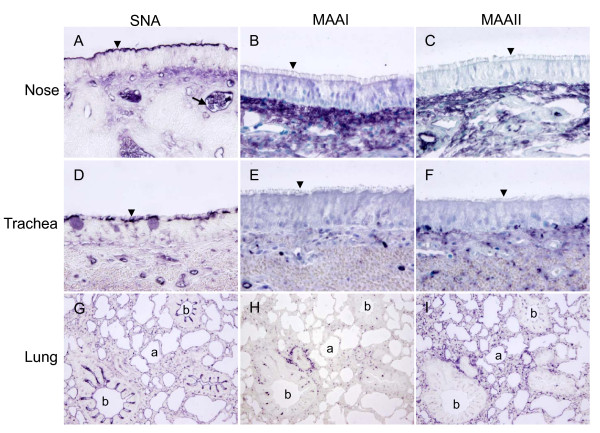
**Lectin staining in nose, trachea, and lung from pigs**. Examples of lectin staining demonstrating infAv receptors SNA lectin (SA-α-2,6-terminal saccharide) and MAAI and II lectin (SA-α-2,3-terminal saccharides), respectively, in nose, trachea, and lung of pigs. The lectin staining appear as a dark blue colour. **A**: Nasal epithelium demonstrating SNA lectin staining. A SNA lectin positive lining of epithelial cells (arrowhead) and SNA positive endothelial cells (arrow) are shown. **B **and **C**: Nasal epithelium stained with MAAI and MAAII lectin, respectively. It is not possible to recognise staining of the surface of the epithelium (arrowhead). **D**: Tracheal epithelium demonstrating SNA lectin staining. Positive staining is shown of the surface of the epithelium (arrowhead). **E **and **F**: Tracheal epithelium stained with MAAI and MAAII lectin, respectively. It is not possible to recognise staining of the surface of the epithelium (arrowhead). **G**: Lung section demonstrating SNA lectin staining. A SNA lectin positive lining of epithelial cells is seen in alveoli (a) and bronchioles (b). **H**: Lung section demonstrating MAAI lectin staining. Very few epithelial cells are positive in both bronchioles (b) and alveoli (a). **I**: Lung section demonstrating MAAII lectin staining, scattered positive reaction is shown in the alveolar epithelial cells (a) and few positive cells are seen in bronchioles (b). (Original magnifications A, B, C, D, E, and F: x40; G, H, and H: × 10).

Both influenza virus positive pigs (n = 8) as well as influenza virus negative pigs (n = 13) had a strong staining of the luminal part of the respiratory epithelial cells with the SNA lectin demonstrating SA-α-2,6-terminal saccharides (Figure 1). There was a coherent signal lining of the SNA lectin at the epithelial cells of nose, trachea, bronchi, bronchioles, and alveoli (Figure 2). Furthermore, endothelia cells were demonstrated to be SNA lectin positive (Figure 2).

There was no differences in the SNA signal of the non-affected lung tissue among the different groups of pigs including the non-inoculated sentinel pig, however, in the consolidated areas of the infAv positive pigs the SNA signal was scarce to non-existing.

**Figure 3:**
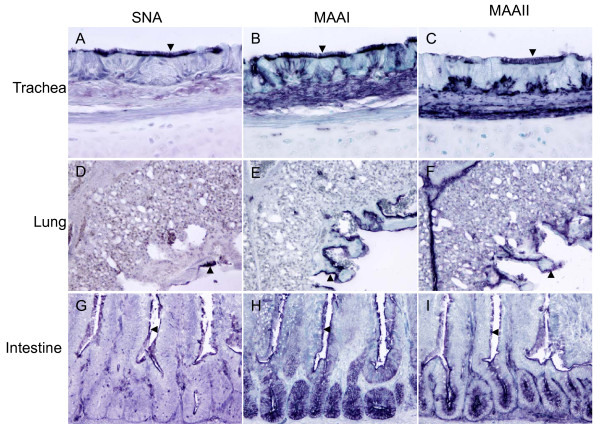
**Lectin staining in trachea, lung, and intestine from chickens**. Examples of lectin staining demonstrating infAv receptors SNA lectin (SA-α-2,6-terminal saccharide) and MAAI and II lectin (SA-α-2,3-terminal saccharides), respectively, in trachea, lung, and intestine from chickens. The lectin stainings appear as a dark blue colour. **A, B**, and **C **demonstrate staining of the luminal surface of the tracheal epithelium by all three lectins SNA, MAAI, and MAAII, respectively, at the epithelium (arrowheads). The MAAI staining (C) is scarcer than the SNA (A) and MAAII (B) staining. **D **demonstrates SNA lectin staining of the lung of chicken, only a small area of positive staining of the epithelium in a parabronchus is seen (arrowhead). **E **demonstrates MAAI staining of the lung of a chicken, luminal staining of the epithelium in a parabronchus is seen (arrowhead). **F **demonstrates MAAII staining of the lung (arrowhead), the staining is less on the epithelium in the lumen of the lung compared to the MAAI staining (E). **G, H**, and **I **are intestinal epithelium demonstrating staining of the luminal surface of the epithelium (arrowheads) by all of the three lectins SNA, MAAI, and MAAII, respectively. (Original magnifications A, B, and C x40; D, E, F, G, H, and I x20).

The epithelial cells in intestines and trachea of chickens were also highly positive for the SNA lectin whereas there was a very sparse signal for epithelial cells of the lungs (Figure 3).

#### SA-α-2,3-terminal saccharides (MAAI lectin)

Only few epithelial cells of the alveoli and bronchioles were positive for the receptor SA-α-2,3-terminal saccharide when the MAAI lectin was used for staining (Figures 1 and 2). The MAAI lectin could not be demonstrated on the epithelial cells of nose and trachea, and only in very few bronchial epithelial cells whereas some cells in lamina propia of the epithelium and endothelial cells were MAAI lectin positive. The distribution of the MAAI lectin was similar in all groups of pigs, both infAv infected and infAv negative pigs. In general, the MAAI lectin staining was scarce, and it seemed that the signal was mainly confined to alveolar type II cells. Similar to the SNA lectin signal, the MAAI staining was not observed in consolidated areas of the infAv positive pigs.

The tissues from chickens were highly positive for the MAAI lectin (Figure 3). Especially the luminal surface of the epithelial cells of intestine demonstrated strong staining for the MAAI lectin, but also ciliated epithelial cells of trachea and epithelial cells of the lung were positive.

#### SA-α-2,3-terminal saccharides (MAAII lectin)

The signal from the MAAII lectin demonstrating the SA- α-2,3-terminal saccharides had a more pronounced individual variation among pigs compared to the MAAI lectin signal. It was not possible to correlate this individual variation to the different groups of pigs. The MAAII lectin was not demonstrable on the epithelial cells of the nose, and trachea (Figure 1). However, MAAII was demonstrated on epithelial cells of the alveoli and bronchioles and very few cells of bronchi (Figure 1). Furthermore, the connective tissue of lamina propria and submucosa were MAAII positive, but the endothelial cells were negative. The MAAII lectin staining was generally more widespread than the MAAI lectin staining and was present on the surface of both alveolar type I and II cells. As for the SNA lectin staining, the MAAII lectin staining was scarce to not present in the consolidated areas of the infAv positive pig lungs.

The tissue from chickens had a high MAAII signal, especially in the intestines (Figure 3). The ciliated epithelial cells of trachea demonstrated a luminal staining for MAAII lectin. However, there were fewer positive cells than for MAAI lectin and, likewise, fewer epithelial cells of the lung were positive. As for the pig, connective tissue in lamina propia of the epithelium and in submucosa was also MAAII positive.

As described above there was a clear difference in the distribution of the three lectins in the consolidated and the non-consolidated areas of the lung. In the non-consolidated areas it was not possible to detect any difference when comparing the different groups of pigs. However, in the consolidated areas of the lung of both AIV and SIV infected pigs, staining with all three lectins were scarce or not present.

### Evaluation of receptor staining

To evaluate the distribution and extent of the receptors SA-α-2,6 and 2,3-terminal saccharides detected with the three lectins SNA, MAAI, and MAAII, blinded scoring was performed by two persons (table 1). The agreement was tested statistically using the weighted kappa test. A weighted kappa coefficient (κ_w_) of 0.73 was obtained. A coefficient of 1 is 100% agreement and the κ_w _= 0.73 is considered a good agreement between scores [27].

**Table 1:** Results from double-blinded scoring of lectin staining

	0-20%	20-40%	40-60%	60-80%	80-100%	Total
0-20%	**34**	6	1	1	0	42

20-40%	0	**4**	6	2	1	13

40-60%	0	0	**1**	2	1	4

60-80%	0	1	0	**0**	4	5

80-100%	0	0	0	1	**12**	13

Total	34	11	8	6	18	77

The table shows the results from double-blinded scoring of lectin staining SNA, MAAI, and MAAII, respectively, in normal unconsolidated areas of the lung sections as well as tracheal and nasal epithelium. The agreement between the two evaluators in the five scoring categories can be read from the table. The indicated scorings were used for calculation of the weighted kappa coefficient.

### Demonstration of infAv antigens

**Figure 4:**
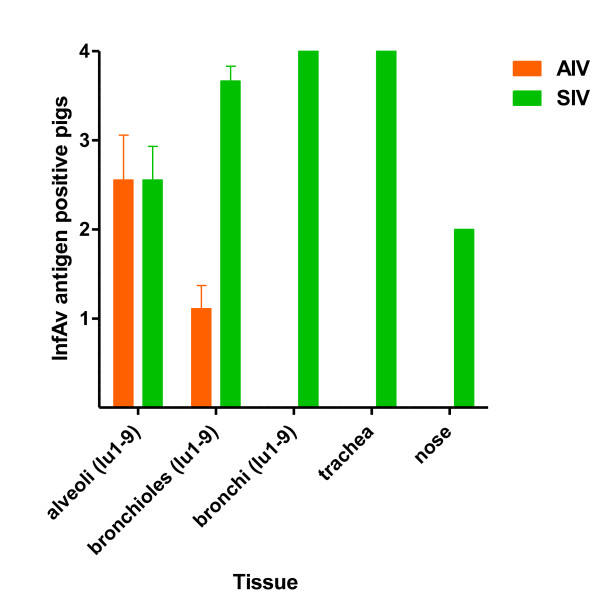
**Distribution of influenza antigens in swine and avian influenza virus infected pigs**. The figure sums up the results from demonstration of influenza A virus (infAv) antigens with immunohistochemistry in alveoli, bronchioles, bronchi as well as nasal and tracheal epithelium. The pigs were inoculated with avian influenza A virus (AIV) H4N6 (n = 4 pigs) or swine influenza A virus (SIV) (n = 4) including two H1N1 and two H1N2 infected pigs. The figure shows the number of infAv positive pigs out of the four pigs in each group (AIV and SIV, respectively). For the alveoli, bronchioles, and bronchi the number of infAv positive pigs is averaged over the 9 lung sections (lu1-9).

The overall result of the infAv antigen staining revealed that the SIV antigens were widespread in the bronchi and bronchioles and in the alveoli of the most severely affected areas. In contrast, the AIV antigens were mainly demonstrated in the alveoli and in a few bronchiolar epithelial cells whereas it was not found in the upper respiratory tract. The results of the infAv antigen staining are summarized in Figure 4 and table 2.

**Table 2:** Results from immunohistochemical staining of influenza A virus antigen

Inoculum Necropsy day		AIV H4N6 PID4		AIV H4N6 PID8		SIV H1N2 PID4		SIV H1N1 PID4		Mock PID4		Mock PID8		Sent. PID8
**Pig**		**A**	**B**	**C**	**D**	**E**	**F**	**G**	**H**	**I**	**J**	**K**	**L**	**M**

	alv	+	+	-	+	-	+	+	-	-	-	-	-	-
	
lu1	br.iol	+*	+	-	+*	-	+	+	+	-	-	-	-	-
	
	bro	-	+*	-	-	+	+	+	+	-	-	-	-	-
	

	alv	+	+	+	+	+	+	+	+	-	-	-	-	-
	
lu2	br.iol	+*	+	-	+	+	+	+	+	-	-	-	-	-
	
	bro	-	+*	-	-	+	+	+	+	-	-	-	-	-

	alv	+	+	+	+	+	+	+	+	-	-	-	-	-
	
lu3	br.iol	+	+	+*	+*	+	+	+	+	-	-	-	-	-
	
	bro	+*	+*	-	-	+	+	+	+	-	-	-	-	-

	alv	+*	-	-	-	+	-	-	+	-	-	-	-	-
	
lu4	br.iol	-	-	-	-	+	+	+	+	-	-	-	-	-
	
	bro	-	-	-	-	+	+	+	+	-	-	-	-	-

	alv	-	+	+	+	+	-	-	+	-	-	-	-	-
	
lu5	br.iol	-	+	+	-	+	+	+	+	-	-	-	-	-
	
	bro	-	-	-	-	+	+	+	+	-	-	-	-	-

	alv	-	+	+	+	i	-	-	+	-	-	-	-	-
	
lu6	br.iol	-	+	-	-	i	+	+	+	-	-	-	-	-
	
	bro	-	+*	-	-	i	+	+	+	-	-	-	-	-

	alv	+	+	+	+	+	+	+	+	-	-	-	-	-
	
lu7	br.iol	-	+	+*	-	+	+	+	+	-	-	-	-	-
	
	bro	-	-	-	-	+	+	+	+	-	-	-	-	-

	alv	+	+	+	+	+	-	-	+	-	-	-	-	-
	
lu8	br.iol	-	+	+*	+*	+	-	+	+	-	-	-	-	-
	
	bro	+*	+*	-	-	+	+	+	+	-	-	-	-	-

	alv	-	-	-	+*	-	-	+	+	-	-	-	-	-
	
lu9	br.iol	-	-	-	-	+	+	+	+	-	-	-	-	-
	
	bro	-	-	-	-	+	+	+	+	-	-	-	-	-

Trachea		-	-	-	-	+	+	+	+	-	-	-	-	-

Nose		-	-	-	-	+	+	-	-	-	-	-	-	-

lu1: cranioventral part of the left cranial lobe; lu2: caudoventral part of the left cranial lobe; lu3: dorsal part of the left cranial lobe; lu4: ventral part of the right cranial lobe; lu5: middle part of the right cranial lobe; lu6: dorsal part of the right cranial lobe; lu7: middle part of the right middle lobe; lu8: middle part of the accessory lobe; and lu9: middle part of the right caudal lobe.

PID = post inoculation day, *Single positive cell demonstrated, i = not done.

Results from immunohistochemical staining of influenza A virus (infAv) antigens in nose, trachea and the nine lunge sections from avian influenza A virus (AIV) H4N6 inoculated pigs, swine influenza A virus (SIV) H1N1 and H1N2 inoculated pigs, the mock-inoculated pigs, and the sentinel (Sent.) pig which was contained in the AIV group. Each lung tissue section was evaluated for infAv antigens in alveoli (alv), bronchioles (br.iol), and bronchi (bro).

#### SIV infected pigs

**Figure 5:**
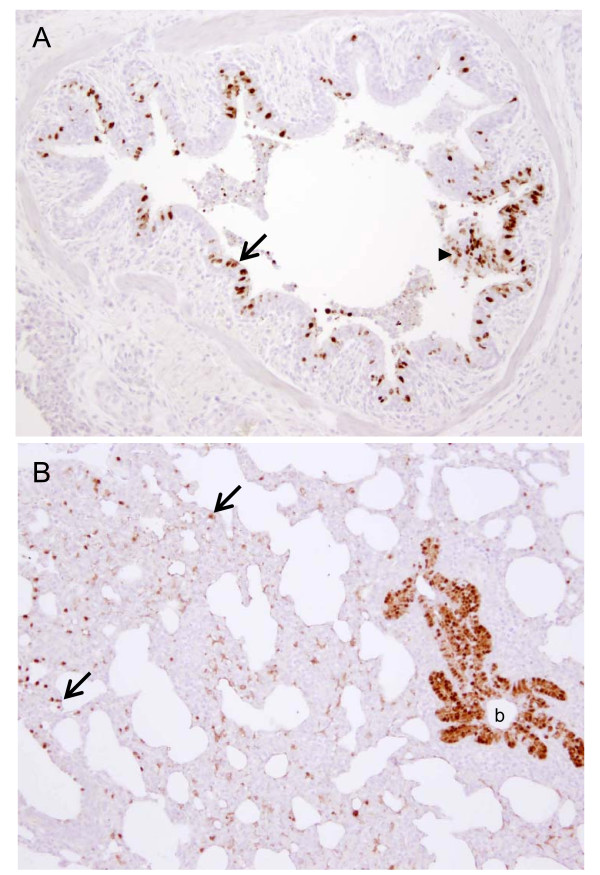
**Immunohistochemical staining of swine influenza virus antigens in lung tissue from a pig**. Immunohistochemical detection of swine influenza virus (SIV) antigens using polyclonal rabbit anti-swine influenza antibody against A/swine/Denmark/4744/1981 (H1N1) and counterstaining with hematoxylin. **A**: Lung section from SIV H1N1 infected pig euthanized PID 4. SIV antigen positive (brown) epithelial cells (arrow) are seen in a bronchus. SIV antigen positive cells are also seen in the exudates in lumen of the bronchus (arrowhead). Original magnification x10. **B**: Lung section from SIV H1N2 infected pig euthanized PID 4. SIV antigen positive (brown) epithelial cells are seen in a bronchiole (b) and in alveolar septa (arrows). (Original magnification x10).

For the SIV infected groups, two different primary antibodies were used for IHC and showed similar results. The nasal and tracheal epithelium had scattered SIV antigen positive cells. In pigs from both the H1N1 and H1N2 infected groups, a lobular distribution of SIV antigen could be demonstrated in the lung sections. A lining of SIV antigen positive epithelial cells were seen predominantly in bronchi and bronchioles of the affected areas (Figure 5 and table 2). The exudates in lumen of affected bronchi and bronchioles were often SIV antigen positive. Furthermore, SIV antigens could sometimes be found in alveolar epithelial cells (Figure 5 and table 2). The H1N2 infected pigs had more SIV antigen positive areas compared to the H1N1 infected pigs.

SIV antigens were present both in the cranial and the caudal lung tissue sections. However, the cranial lung lobes were most severely affected, especially the left cranial lobe. There was a clear correlation between areas of consolidated lung tissue and presence of SIV antigen positive cells.

#### AIV H4N6 infected pigs

**Figure 6:**
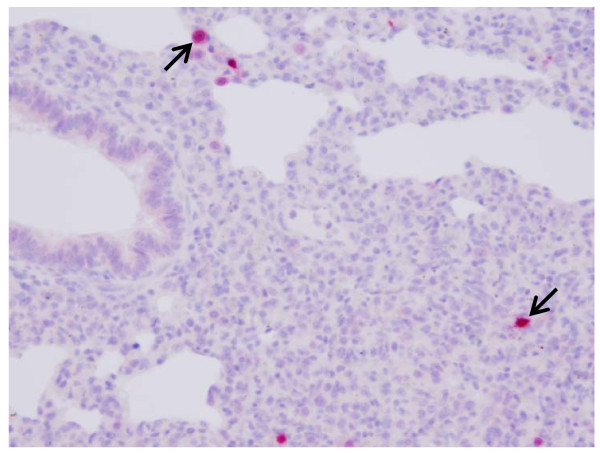
**Immunohistochemical staining of avian influenza virus antigens in lung tissue from a pig**. Immunohistochemical detection of avian influenza virus (AIV) antigens in lung section from AIV infected pig euthanized PID 4, using goat anti-influenza A antibody and counterstaining with hematoxylin. AIV antigen positive (red) epithelial cells are seen in the alveolar septa (arrows). The area is partly consolidated. (Original magnification x20).

The nose and trachea were AIV antigen negative in the pigs infected with AIV H4N6 (table 2). In the bronchi, only a single positive cell was seen in a few lung sections from pigs euthanized PID 4. AIV antigens were demonstrated in alveoli and in some bronchioles (Figure 4, 6 and table 2) of all examined lung lobes except in the right caudal lobe. The cells demonstrating AIV antigens were few and sporadically spread in the tissues (Figure 6). Similar to SIV infected pigs, the highest number of AIV antigen positive cells was seen in the left cranial lobe. In general, the number of AIV antigen positive cells was very low compared to the number of infAv antigen positive cells found in the SIV infected pigs. In general, the pigs which were euthanized PID 8 had less AIV antigen positive cells than the pigs euthanized PID 4.

#### Control pigs

InfAv antigens could not be demonstrated in mock-inoculated pigs (table 2) or in the pigs that had recovered from SIV infections 8 weeks earlier. The sentinel pig in the AIV inoculated group were also infAv antigen negative (table 2).

### Double-staining of infAv antigen and cytokeratin

**Figure 7:**
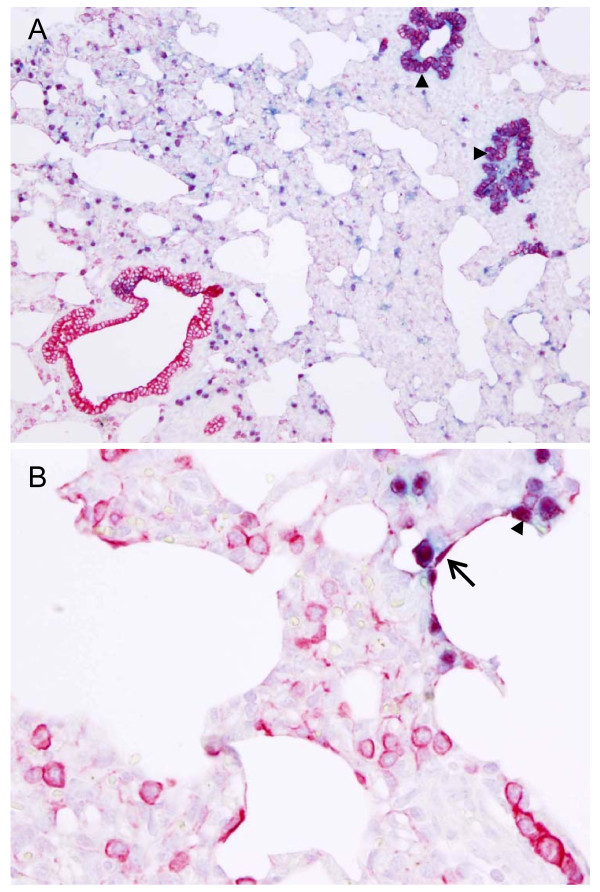
**Double immunohistochemical staining of swine influenza virus antigens and epithelial cells**. Lung sections from swine influenza virus (SIV) H1N1 infected pig euthanized PID 4. Double immunohistochemical staining using polyclonal anti-influenza and anti-cytokeratin antibodies demonstrating SIV antigen positive cells (clear blue cells) and epithelial cells (clear red cells). Double positive cells (SIV antigen positive epithelial cells) are purple (counterstained with hematoxylin). **A: **Demonstration of double positive bronchioles (arrowhead). **B: **Demonstration of both double stained alveolar type I (arrow) and II epithelial cells (arrowhead). (Original magnifications A: x10, B: x40).

Double positive cells, stained for both infAv antigen and cytokeratin, were found in the alveoli of the SIV infected pigs, demonstrating that the SIV antigen positive cells were alveolar epithelial cells. Furthermore, based on the morphology of the infAv antigen positive epithelial cells, it was possible to conclude that both type I and II epithelial cells were SIV antigen positive demonstrating that SIV infects both types of cells (Figure 7). Epithelial cells in several of the bronchi and bronchioles also showed a double staining, demonstrating bronchi and bronchioles as predilection sites for SIV in these areas of the respiratory tract.

**Figure 8:**
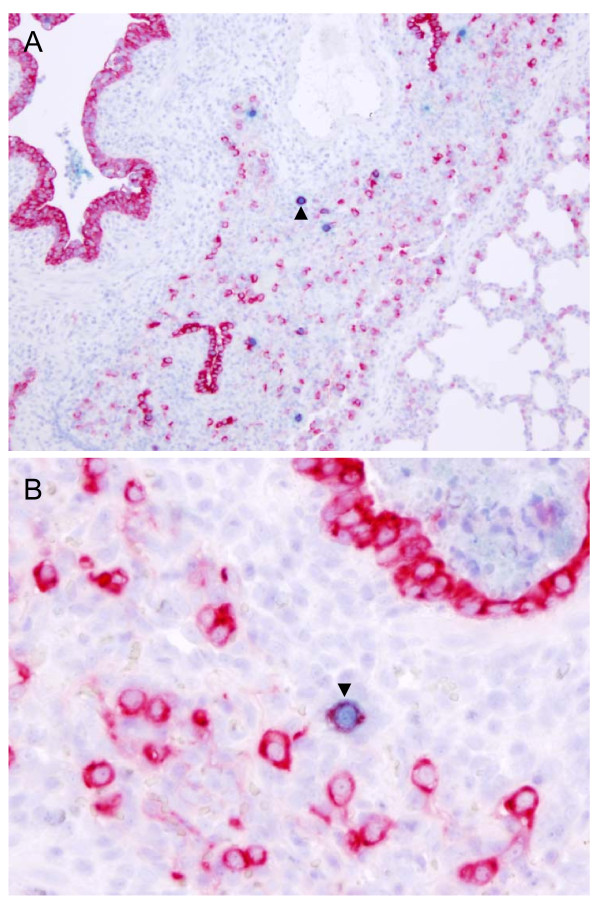
**Double immunohistochemical staining of avian influenza virus antigens and epithelial cells**. Lung sections from avian influenza virus (AIV) infected pig, euthanized PID 4. Double immunohistochemical staining using polyclonal anti-influenza and anti-cytokeratin antibodies (counterstaining with hematoxylin) demonstrating AIV antigen positive cells (clear blue cells) and epithelial cells (clear red cells). Double positive cells (AIV antigen positive epithelial cells) are purple. **A: **Demonstration of double positive alveolar epithelial cells (arrowhead). **B: **Greater magnification than A, demonstration of double stained alveolar type II epithelial cell (arrowhead). (Original magnifications A: x10, B: x40).

The double-stainings of the tissues from AIV infected pigs clearly verified that the large infAv antigen positive cells found in the alveoli were epithelial cells. Furthermore, based on the morphology, it was possible to demonstrate that AIV antigens were present only in alveolar type II epithelial cells (Figure 8) in contrast to SIV antigens which were found in both alveolar type I and II epithelial cells (Figure 7).

For both double staining protocols (respectively AIV and SIV antigen demonstration), there was no difference in the number of infAv antigen positive cells in comparison to the infAv antigen single staining protocols. Furthermore, the double staining protocols showed the same number of positive cells when omitting either the infAv antibodies or the cytokeratin antibody.

### Receptor distribution in consolidated areas

**Figure 9:**
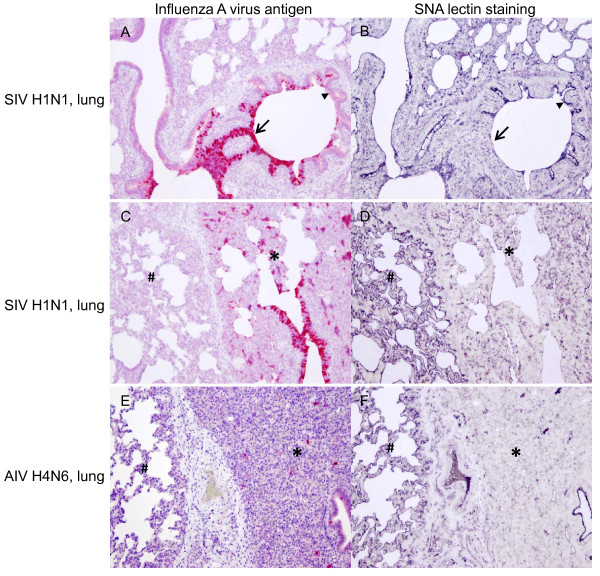
**Comparison of influenza virus infected and non-infected lung tissue and lectin staining**. **A: **Bronchiole and lung parenchyma from swine influenza virus (SIV) H1N1 infected pig euthanized PID 4. SIV antigen positive epithelium (red) is demonstrated by immunohistochemistry using polyclonal goat anti-influenza A antibody and counterstaining with hematoxylin. In the same section it is possible to identify infected (arrow) and non-infected (arrowhead) bronchiolar epithelium. **B: **Parallel section to A demonstrating SNA lectin staining (dark blue) of the SA-α-2,6-terminal saccharide receptor. The lectin staining in the non-influenza infected part of the bronchiole (arrowhead) is considerable stronger compared to the infected part (arrow). **C**: Lung section from SIV H1N1 infected pig euthanized PID 4. Influenza positive cells (red) are demonstrated by immunohistochemistry using polyclonal goat anti-influenza A antibody and counterstaining with hematoxylin. In the same section it is possible to identify an infected (*) and non-infected lobules (#). **D**: Parallel section to C demonstrating SNA lectin staining. The lectin staining is reduced in the influenza infected area (*). **E**: Lung section from avian influenza (AIV) infected pig euthanized PID 4 stained for AIV antigens (red). In the same section it is possible to identify an infected (*) and non-infected lobulus (#). **F**: Parallel section to E demonstrating SNA lectin staining. A reduction of lectin staining is seen in the influenza infected lobulus (*) compared to the non-infected lobulus. (Original magnifications A, B, C, and D x10).

To compare the distribution of the lectin binding sites with the distribution of infAv antigen positive cells, lectin staining and IHC were performed on parallel sections. Interestingly, for both AIV and SIV infected animals it was demonstrated that infAv antigen positive areas lacked lectin binding indicating that the receptors were modified or not expressed in areas with infAv infected cells (Figure 9). It was especially clear when comparing lung sections from the SIV infected pigs where clear infAv antigen positive bronchioles were free from lectin staining in parallel sections (Figure 9). In the AIV infected pigs where the infAv antigen signal was demonstrated in scattered cells a direct comparison was more difficult, however, it was possible to distinguish the same areas of lobuli in parallel lectin stained sections which clearly were without staining (Figure 9). The finding was seen in stainings with all three lectins.

## Discussion

The present study revealed several interesting findings regarding infAv receptors and infAv distributions in pigs. The SA-α-2,6-terminal saccharide receptor was found to be present in high amounts in all areas of the respiratory tract of all examined pigs regardless of their infection status. In contrast, the SA-α-2,3-terminal saccharide receptor was present only on the luminal surface of bronchiolar, but mostly alveolar epithelial cells in the lower respiratory tract.

Most human and swine influenza A viruses show highest affinity for SA-α-2,6-terminal saccharide receptors while most avian influenza A viruses show highest affinity for SA-α-2,3 receptors [9,13-16,19,29]. Mapping of the distribution of receptors in the respiratory tract of different infAv hosts is therefore highly relevant for clarifying host susceptibility to various infAv strains. Earlier studies of the respiratory tract of humans have shown that humans predominantly possess the SA-α-2,6 receptor and ducks expresses mainly the SA-α-2,3 receptor [30,31]. However, humans also possess SA-α-2,3 in the lower part of the respiratory tract and domestic birds like chicken and quail have also been shown to express both receptors [26,31-33]. In our study we were able to confirm that chickens possess SA-α-2,6 receptors in rather large amounts in intestines, trachea and to a lesser extent in lungs.

Previous studies have reported that pigs have both the SA-α-2,6 and the SA-α-2,3 receptors in the trachea and therefore pigs have been regarded as the primary mixing vessel for new infAv subtypes [8,9]. In the present study we could not detect the SA-α-2,3-terminal saccharide in the upper respiratory tract of pigs. Our findings are in accordance with the results of recent studies by Nelli et al. [18] and Van Poucke et al. [17], the latter examined *ex vivo *explants of tissue from the respiratory tract of pigs and found the same receptor distribution in pig tissue as we did. The findings are also in concordance with similar studies in AIV infected humans showing that it is mostly the lower areas of the lung that are infected with AIV, resulting in pneumonia [33-35]. Taken together, these recent studies indicate that the "pig as mixing vessel" theory is less substantial than thought previously and that other species such as chickens and even humans could act as mixing vessels [26,31,32].

There are several possible explanations for the discrepancies in receptor distribution found in different studies. One obvious source of variables is the choice of lectins used for the receptor staining. Thus, Nicholls et al. [23] showed how lectins from different manufacturers may vary in specificity and sensitivity. In addition, both isoforms of MAAI and II recognise SA-α-2,3, but are different in the way they recognises the inner sugar structures. In order to detect all SA-α-2,3 receptors it is important to use both isoforms. Furthermore, since lectins are ubiquitously distributed in different tissues and constitute a component of mucus it is important to be able to differentiate respiratory epithelial cells from other lectin signal positive components in the respiratory tract. We have tried to meet these uncertainties in our scoring system by using relatively wide percentage ranges of the distribution in staining and by specifically evaluating the epithelial cells throughout the respiratory tract. This allowed us to identify the non-specific staining. Furthermore, the selection of pigs for the study may also be important since the receptor distribution may be dependent on various factors such as age, infection status, pig breed etc. However, this has not been documented.

Factors other than the distribution of receptors, may affect the host specificity of a given infAv [20,33] and it has been shown that some infAv's are able to adapt to a given host species resulting in a change in the receptor specificity during infection [20,36,37]. However, the receptor recognition is a crucial step for an infecting virus and therefore it has a large impact on the successful attachment and further replication of virus.

It is likely that the risk of infection is dependent on both the amount of receptors present on the site of infection and the infection dose. In our experimental conditions very heavy exposure will of course increase the chance of infection. This is also seen in human infections with H5N1 where the majority of the infected individuals were exposed to high doses of infAv, probably allowing some of the viruses to enter the lower areas of the respiratory tract where the receptor preferred by the avian viruses is expressed [38,39].

One of our observations was that endothelial cells were SNA and MAAI lectin positive. This finding has also been observed in receptor studies in humans [33]. InfAv's predominantly infect the respiratory epithelial cells and are therefore not in contact with endothelial cells. However, in a damaged epithelium surface infAv may get in contact with endothelia cells. It is, however, most likely that other cell factors such as the availability of specific proteases impact the success of infecting e.g. the endothelia cells.

Interestingly, in parallel sections comparing infAv antigen positive cells with lectin staining it was demonstrated that the lectin staining was markedly diminished in areas where infAv antigen positive cells were present. A likely explanation could be that the infAv neuraminidases have cleaved off the sialic acids during the infection. Afterwards there could be a delay in re-establishing the receptors on infected cells or it could be a defence mechanism prompted by the host cell. To our knowledge this has not been shown earlier but is interesting because it would significantly impact the risk of a single cell being infected with different infAv subtypes and thereby reduce the risk of generating re-assorted viruses. Further studies should be performed on this issue.

The two different SIV isolates used in the present study were found to infect nose, trachea, bronchial, bronchiolar and alveolar type I and II epithelial cells, but the infections were mainly located in the bronchial epithelial cells. The distribution of the SIV antigen positive cells had a lobular distribution for both H1N1 and H1N2 infected pigs, but more lobules were affected in H1N2 infected pigs. The lung lesions and the distribution of SIV found in this study were similar to other SIV infection studies [40-44].

Compared with the SIV infected pigs, only limited infection with very few affected areas was seen in pigs infected with the avian H4N6 strain and the infection with AIV was confined to the lower respiratory tract where especially the alveolar type II epithelial cells, but also a few epithelial cells of bronchioles were infected. Low-grade infection after experimental inoculation with AIV in pigs is in agreement with other studies comparing AIV and SIV infections in pigs [45,46]. The predilection of an AIV for swine alveolar type II alveolar cells has to our knowledge not been described earlier, but alveolar type II cells have been found to be the primary cell type infected in the lungs of fatal human cases of HPAI H5N1 AIV infections [34,35,47]. This may indicate a preference of AIV for alveolar type II epithelial cells in both pigs and humans. Our study indicates that there is a predominance of SA-α-2,3-terminal saccharides on the alveolar type II epithelial cells compared to type I cells and this could explain why the avian virus is confined to alveolar type II epithelia cells. This also opens the possibility of using the pig as a model for the study of the pathogenesis of AIV infection in humans.

## Conclusion

The present study showed that the distribution of the two different infAv receptors in pigs were similar to that of humans, that AIV infection of pigs are confined to the lower respiratory tract and that AIV has a predilection for alveolar type II epithelial cells. Finally, the study revealed that the infAv receptors are modified or absent in SIV and AIV infected cells.

## Competing interests

The authors declare that they have no competing interests.

## Authors' contributions

RT participated in the conception and design of the study, carried out the experiments including the subsequent analysis and interpretation of data, as well as drafted the manuscript. LEL participated in the conception and design of the study, analysis and interpretation of data and revision of the manuscript. BMV participated in the conception and design of the study, analysis and interpretation of data, as well as revision of the manuscript. All authors read and approved the final manuscript.
